# Experiences of social prescribing in the UK: a qualitative systematic review

**DOI:** 10.3399/BJGP.2024.0179

**Published:** 2025-02-11

**Authors:** Nur Hidayati Handayani, Marta Wanat, Stephanie Tierney

**Affiliations:** Nuffield Department of Primary Care Health Sciences, University of Oxford, Oxford.; Nuffield Department of Primary Care Health Sciences, University of Oxford, Oxford.; Nuffield Department of Primary Care Health Sciences, University of Oxford, Oxford.

**Keywords:** general practice, link workers, mental health, qualitative research, social prescribing, systematic review

## Abstract

**Background:**

Social prescribing connects patients to resources or activities to meet their non-medical needs. In the UK, it is often implemented in primary care. In the social prescribing pathway, patients are directed to link workers to identify suitable solutions for their needs such as art workshops or welfare benefit guidance. Social prescribing marks a notable transition from traditional medical treatments to more comprehensive strategies focusing on holistic health and wellbeing. Insights from patient experiences can improve the development of social prescribing to better meet their needs. This understanding can aid in improving the delivery and outcomes of social prescribing.

**Aim:**

To synthesise qualitative research on the experiences of social prescribing among patients in the UK.

**Design and setting:**

Qualitative systematic review using thematic synthesis for peer-reviewed studies that focused on experiences of users of social prescribing in the UK.

**Method:**

An exhaustive search was performed in six databases: ASSIA, CINAHL, Embase, MEDLINE, PsycINFO, and Social Sciences Citation Index via Web of Science. The Critical Appraisal Skills Programme tool for qualitative research was used for quality assessment and the PRISMA 2020 checklist was used to ensure the report transparency.

**Results:**

Titles and abstracts of 1269 studies were screened. In total, 85 studies were full-text screened, and 19 studies were included in the review. Five analytical themes were developed from these studies: a) searching for hope in times of adversity; b) variability in temporal responsiveness; c) sustained change from a positive response; d) feeling supported and empowered by the social prescribing pathway; and e) misalignment producing no response.

**Conclusion:**

Patients might experience lasting advantages from social prescribing if it aligns with their needs and expectations. Results highlighted the importance of matching social prescribing referral with patients’ readiness to engage. Therefore, it is recommended that healthcare professionals evaluate patient suitability before beginning a social prescribing referral.

## Introduction

Social prescribing aims to improve health and wellbeing through connecting individuals to non-clinical services to address their non-medical needs (for example, loneliness or housing instability).^1^^–^3 It seeks to address the multifaceted determinants of an individual’s health.^3^^,^^4^ Link workers play a key role in delivering social prescribing, taking time to help patients identify their non-medical needs and directing them to relevant community support. Across the UK, social prescribing has been incorporated into various healthcare settings such as general practices, community health centres, and NHS trusts.^5^ In England, the NHS has integrated social prescribing into primary care by assigning link workers in primary care networks.^6^

Patients who have received social prescribing may experience increased self-esteem, strengthened social connections, and acquire new skills and interests.^7^ Questions remain regarding its effectiveness in improving health outcomes and healthcare service utilisation.^7^^–^9 Nevertheless, social prescribing is seen as a promising strategy to overcome health inequities through partnerships between primary care and the third sector, which includes non-profit organisations, charities, and voluntary groups that provide community-based support.^7^

Research using a qualitative approach on this topic is rapidly growing but, to the authors’ knowledge, it has not been synthesised. This could help with developing a deeper and more conceptual understanding of patients’ experiences. Hence, a qualitative systematic review was conducted that aimed to bring together research on patients’ experiences with social prescribing in the UK. It sought to understand both positive and challenging experiences, focusing on interactions between patients and primary stakeholders such as link workers and GPs. The aim was to provide recommendations for service and policy improvements and to identify areas needing further research.

**Table:** How this fits in

Social prescribing is proposed to enhance patient outcomes by addressing social determinants of health. This is the first qualitative systematic review, to the authors’ knowledge, to explore patient experiences with the social prescribing pathway in the UK. The findings highlight the varying ways that patients may respond to social prescribing, which can be shaped by their readiness and capacity to engage. Clinicians can use this information to tailor their approaches, advocate for patient-centric care, and improve accessibility.

## Method

Qualitative systematic reviews are a structured approach to comprehensively search, evaluate, and merge findings from qualitative research.^10^ Thematic synthesis was used as an approach for synthesising the data.^11^ A review protocol was published.^12^

**Figure 1.**
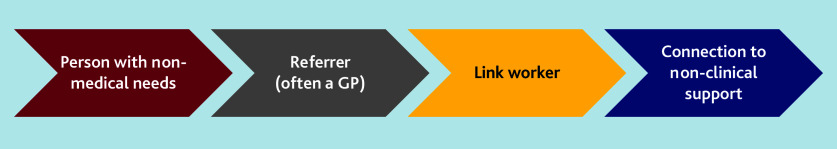
The social prescribing pathway adapted and inspired under the Open Access CC BY 4.0 licence from Hazeldine *et al*.^13^

Peer-reviewed qualitative studies focused on first-hand experiences of users of social prescribing in the UK aged ≥18 years were included. The full social prescribing pathway^13^ had to be covered in studies, meaning that there was a referrer (often a GP), a link worker (other terms may be used, for example, social prescriber), and non-clinical support was suggested or provided (Figure 1).

Studies on service providers’ experiences were excluded unless patient experiences were separately detailed. Studies not written in English were also excluded.

### Searches

Support with the search was provided by a librarian. The search was conducted on the following databases that cover a mixture of health and social sciences literature: ASSIA, CINAHL, Embase, MEDLINE, PsycINFO, and Social Sciences Citation Index via Web of Science. Searches were conducted between 20 October and 5 November 2022. For further information about the MeSH search strategy see Supplementary Box S1.

Forward citation tracking and citation searching of studies included in the review were undertaken, using Citationchaser,^14^ to identify additional studies. A Google alert was also set up to keep abreast of studies completed/published from September 2022 to July 2023. No time limit for the publication date was applied and all references were entered into Covidence (computer software for systematic reviews). Searches were re-run on 13 June 2023 to identify any further studies since the initial searches were conducted.

### Selection process

All titles and abstracts were screened by the first author and a second reviewer (see acknowledgements) to assess eligibility, recording decisions, and reasons for exclusion. When clarity was lacking or references were deemed relevant, full texts were retrieved for further review. Both the first author and the second reviewer (see acknowledgements) examined the full texts of all records progressing from the initial screening for eligibility. In cases of disagreements, the joint senior authors were consulted to reach consensus.

### Data collection

The first author extracted data from the final selected studies into a Microsoft Excel file. The Joanna Briggs Institute Qualitative Data Extraction Tool^15^ was used to guide what data were extracted.

### Quality assessment of the studies

Quality appraisal was performed by the first author using the Critical Appraisal Skills Programme (CASP) tool^16^ for qualitative research. This tool centres on the research method(s) used, data collection techniques, ethical considerations, and the value of the research in addressing the research question.^17^ Appraisal identified recurring quality issues, thereby highlighting improvements for future research.^18^ If ‘yes’ is not answered for the first two or three questions, the evidence may be of poor quality.^19^ All studies were included in the review for their valuable contributions and insights to the qualitative synthesis, and to gather a wide range of perspectives and experiences for a thorough understanding.^20^

### Data synthesis

The first author read all included studies, then transferred them into NVivo to be coded inductively on a line-by-line basis. The first author generated 240 initial codes from included studies (see Supplementary Table S1). These were refined and merged, after discussion as a review team, into 79 codes, which were then grouped together based on commonalities or patterns. Grouping these codes created broader categories or ‘descriptive themes’. Procreate (digital drawing software) was used to craft a visual mind map of how these codes were related. For example, codes ‘patient felt empowered after interaction with link worker’, ‘link worker gave emotional support’, and ‘patients co-created solution to solve their issues with link worker’ were grouped under a descriptive theme ‘strong relationship with link worker’.

After re-examining the 19 included studies to better understand their context, the first author developed a mind map grouping descriptive themes into broader analytical themes. These diagrams provided a visual representation of the analytical themes. Consultations with the wider team were integral during this phase to ensure the synthesised themes were robust and accurate (see Supplementary Table S2, and for an illustration of analytical themes development see Supplementary Figure S1).

The PRISMA 2020 checklist^21^ was used to enhance the transparency of systematic study reporting.

## Results

**Figure 2.**
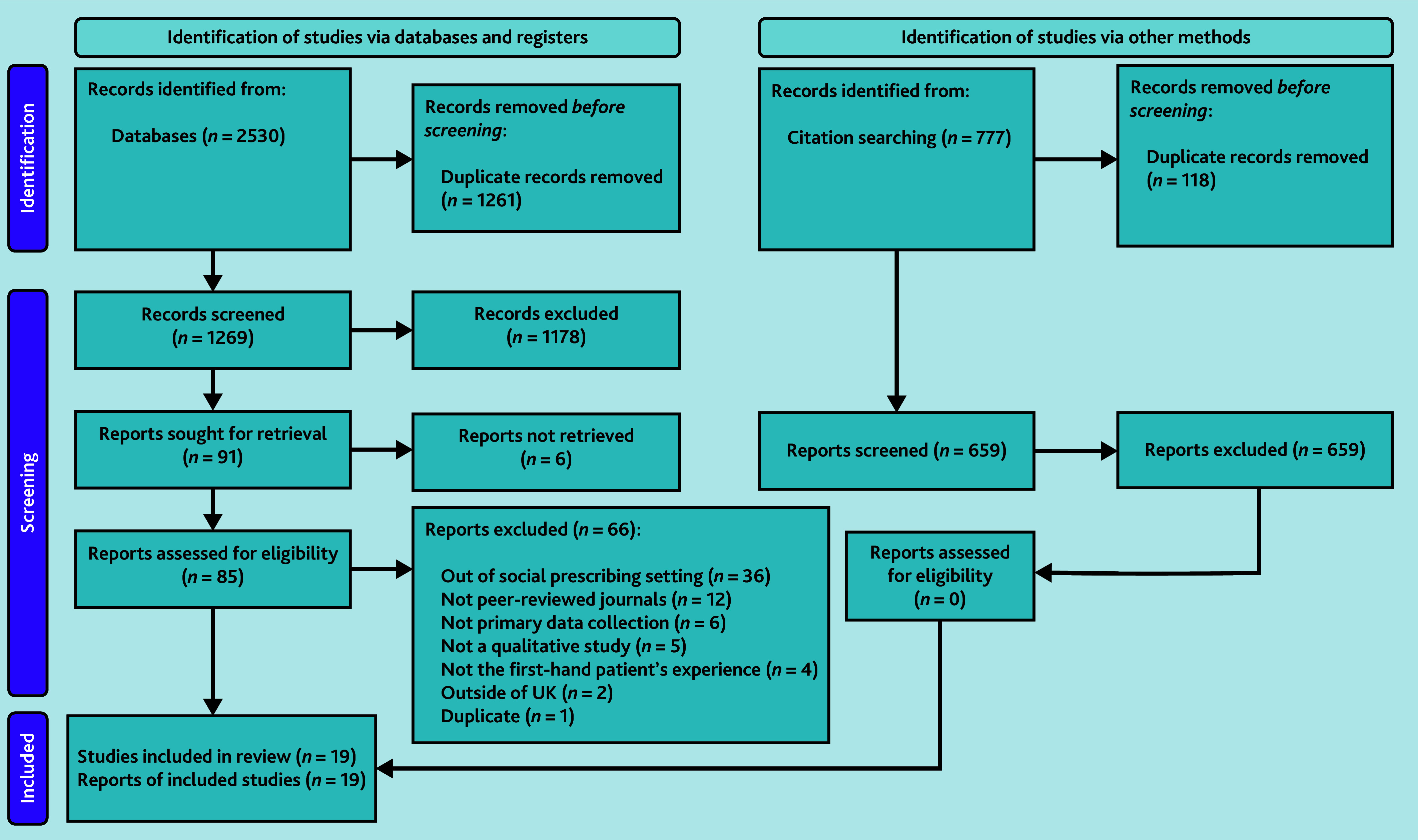
PRISMA chart using format from Page *et al*.^21^

The initial search retrieved 2530 references. After removing duplicates, 1269 references were screened as titles/abstracts for inclusion, from which 91 were sought for retrieval as full text. However, six studies could not be located despite efforts to retrieve them as full texts (for example, contacting authors). Of the 85 studies retrieved, read, and considered, 66 were excluded because they did not cover the social prescribing pathway (*n* = 36), were not in peer-reviewed journals (*n* = 12), did not involve primary data collection (*n* = 6), were not qualitative studies (*n* = 5), did not reflect first-hand patient experiences (*n* = 4), were conducted outside the UK (*n* = 2), or were duplicates (*n* = 1). Forward citation tracking and citation searching of studies included in the review generated 777 records. After 118 duplicates were removed, 659 studies were screened by title and abstract; none were eligible for full-text review. Thus, 19 studies were included in this review. Figure 2 illustrates the screening and selection process.

### Characteristics of included studies

All studies were published between 2017 and 2022 and included a total of 365 people who used social prescribing.^22^^–^40 Sixteen studies were conducted in England,^22^^,^^23^^,^^25^^–^27^,^^29^^–^34^,^^36^^–^40 one in Scotland,^28^ one in the UK,^24^ and one did not specify the study location.^35^ Studies collected data using interviews,^22^^–^36^,^^38^^–^40 as well as ethnographic observation and fieldwork,^26^ observation,^25^ and a combination of interviews and surveys.^38^ Focus group discussions were also used in some studies.^29^^,^^37^ Most of the studies analysed the data thematically,^22^^,^^27^^,^^29^^–^38^,^^40^ narrative and reflective thematic analysis,^23^ interpretative thematic analysis,^24^ thematic analysis with self-determination theory as framework.^28^ Table 1 presents the characteristics of the 19 included studies for this review.

**Table 1.** Characteristics of included studies

**Authors (year)**	**Location**	**Sample size, participants, *n***	**Socioeconomic status of areas of studies (as described by the authors)**	**Data collection method**	**Analysis**
**Carnes *et al* (2017)^22^**	England	20	Mixed socioeconomic (socioeconomic deprivation and affluent area)	Interview	Thematic analysis
**Frerichs *et al* (2020)^23^**	England	19	Not specified	Interview	A narrative analysis and reflective thematic analysis
**Foster *et al* (2020)^24^**	UK	26	Not specified	Interview	Interpretive thematic analysis
**Gibson *et al* (2021)^25^**	England	19	High socioeconomic deprivation area	Interview and observation	Grounded theory
**Gibson *et al*(2022)^26^**	England	19	High socioeconomic deprivation area	Ethnographic observation, interview, and fieldwork note	Grounded theory
**Giebel *et al* (2020)^27^**	England	13	Disadvantaged area	Interview	Thematic analysis
**Hanlon *et al*(2019)^28^**	Scotland	12	High socioeconomic deprivation area	Interview	Thematic analysis using self-determination theory as a framework
**Hassan *et al* (2020)^29^**	England	18	Disadvantaged area	Interview and focus group discussion	Thematic analysis
**Kellezi *et al* (2019)^30^**	England	19	Across the socioeconomic spectrum, living in a relatively affluent area	Interview	Thematic analysis
**Lloyd-Evans *et al* (2020)^31^**	England	19	Mixed socioeconomic (socioeconomic deprivation and affluent area)	Interview	Thematic analysis
**Moffatt *et al* (2017)^32^**	England	30	Socioeconomic deprivation area	Interview	Thematic analysis
**Morris *et al* (2022)^33^**	England	44	Socioeconomic deprivation area	Interview	Thematic analysis
**Nadal *et al* (2022)^34^**	England	6	Diverse socioeconomic backgrounds area	Interview	Thematic analysis
**Payne *et al* (2020)^35^**	Not specified in the UK	17	Socioeconomic deprivation area	Interview	Thematic analysis
**Pescheny *et al* (2018)^36^**	England	10	High socioeconomic deprivation area	Interview	Thematic analysis
**Simpson *et al* (2021)^37^**	England	17	Not specified	Focus group discussion	Thematic analysis
**White *et al* (2022)^38^**	England	7	Not specified	Interview and survey	Thematic analysis
**Wildman *et al* (2019)^39^**	England	24	Socioeconomic deprivation area	Interview	Grounded theory
**Woodall *et al* (2018)^40^**	England	26	Not specified	Interview	Thematic analysis

### Quality appraisal

Results from the CASP tool are presented as supplementary material (see Supplementary Table S3). All included studies scored at least seven out of 10 on the CASP checklist, indicating good quality. Studies primarily lost points because they did not specify the type of relationship between the researcher and the participants.

### Data synthesis

Data highlighted how patients often began their social prescribing experience in search of hope during times of adversity by seeking advice from a GP. Then, their experiences with social prescribing showed variability in temporal responsiveness; patients may have an immediate or more protracted positive response to social prescribing, or misalignment producing no response. In terms of variability in temporal responsiveness, patients received supportive encouragement from both link workers and the community services/groups they accessed, which allowed them to potentially have sustained change.

**Figure 3.**
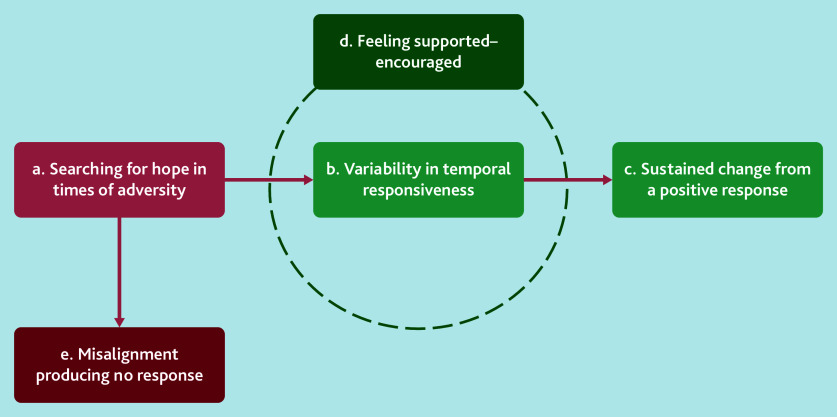
Analytical themes of patients’ experiences of social prescribing in the UK.

Figure 3 illustrates the five analytical themes developed from the synthesis, which underpin this process of seeking support during adversity. It begins with individuals searching for hope (‘a’). Feeling supported and encouraged (‘d’) plays a crucial role, but individuals can vary in temporal responsiveness (‘b’). Support from a link worker can lead to sustained change (‘c’) by reinforcing commitment to long-term positive behaviours. Conversely, misalignment (‘e’) results in no response, highlighting the importance of tailored support for effective social prescribing outcomes (for examples of themes, codes, and quotations, see Supplementary Box S2). These five analytical themes are described in further detail below.

### Searching for hope in times of adversity

Patients were searching for hope to improve their situation, even while facing significant adversity in their lives.^23^^,^^25^^,^^26^^,^^28^^,^^34^^,^^35^ As patients became more conscious of their problems, their immediate action tended to involve seeking assistance from a GP. Some patients faced mental health issues, marked by feelings of vulnerability and a conflict between wanting to be alone and needing real connections:

*‘One question was: “Do I need help?” And my answer was: “Definitely yes!” And it all sort of started from there really.’*^36^

Differences in patients’ social support networks, shaped by socioeconomic status, had an impact on their experiences and results within the social prescribing pathway, and whether they were ready to engage with it.^22^^,^^25^^,^^26^^,^^31^^,^^36^^,^^37^^,^^39^ Patients with good support systems often received encouragement, understanding, and logistical help, making the social prescribing journey more manageable:

*‘While being at work amplified his mental health issues, when Andy returned to work his employer’s flexibility played a central role in enabling his engagement with the intervention and related referrals.’* 25

In contrast, patients lacking support struggled to improve owing to stressful living environments, such as domestic burdens or poverty, often linked to lower socioeconomic status, leading to feelings of isolation, discouragement, and logistical difficulties in attending sessions.

### Variability in temporal responsiveness

Participants varied in how quickly they reported social prescribing had made a difference to their lives. Their reactions and benefits were not uniform; although some experienced immediate improvements in their situation, others noticed changes only after a prolonged period or required tailored support to engage in social prescribing. Based on the synthesis, all patients reported improvements in various aspects when social prescribing matched their needs.^23^^–^38^,^^40^

The immediate benefits and positive changes occurred when patients, soon after participating in recommended activities or interventions, experienced an improvement in their situation.^23^^,^^25^^–^28^,^^30^^–^37^,^^39^^,^^40^ This was influenced by an initial positive interaction with those delivering community services or support, feeling heard and valued, effective matching of patients to activities by link workers, and a welcoming atmosphere in the social prescribing activity:

*‘Doing something like the* [artistic] *course, I could feel the difference already because there are people there of a like mind, people that are creative.’*^23^

On the other hand, patients experienced delayed impact^22^^,^^23^^,^^25^^,^^27^^,^^29^^,^^31^^–^40 caused by several factors: inadequacy of information about social prescribing, poor relationship with the link worker, limited financial resources, and duration of social prescribing. Rushing to signpost patients to activities resulted in them feeling disappointed.^9^ For patients struggling with severe mental health issues and low self-esteem, they required proactive link workers and an adaptable duration of the social prescribing pathway:

*‘I think it probably could have been longer. I think it should be more like help until they think they are done. Cos when I first met her I was really down, but towards the end I was much better but I still could have done with one or two more.’*^40^

### Sustained change from a positive response

Patients experienced sustained improvements when social prescribing activities matched their needs and enabled them to develop skills to cope with challenges:^23^^–^38^,^^40^

*‘She would try and get me to use the phone, but I used to panic … I did eventually do it myself. She wrote things down for me to say on the phone, for me to explain.’*^31^

*‘I would not have been able to do it if I hadn’t have gone to Life Rooms* [referred to by the link worker]*, it give me coping mechanisms, it’s given me strategies, and its helped me to get the confidence and self-esteem because I was at rock bottom.’*^29^

Changes experienced by patients could manifest as improved health/healthier lifestyle, more social engagement, better mental health, increased self-confidence, and securing employment:

*‘I think it’s changed my life completely … I was too fat. I had issues. Ways to Wellness has swept some of them away. It’s been a very, very positive experience … I’m a happy bunny.’*^39^

Positive outcomes could extend beyond the person, fostering community resilience.^25^^,^^27^^,^^28^^,^^35^^,^^40^ Empowered and interconnected individuals sometimes contributed to stronger community unity and cooperation; support from social prescribing inspired a ripple effect of giving back among patients, either to start their own group or to volunteer. This highlighted cyclical support: receiving help and then transitioning into roles that aided others, underscoring the transformative power of community-centric interventions:

*‘Participants felt positive about their experiences of the support provided, which in turn helped them to help others through volunteering. Although none of the participants in this study became volunteer community champions, some did become volunteers at the shop; the very community-based support shop they first accessed for help.’*^27^

### Feeling supported–encouraged

Patients experienced empowerment and support through social prescribing. Link workers’ guidance and encouragement, alongside a non-judgemental attitude and willingness to adapt plans, prompted patients to try new activities and approaches:^23^^–^25^,^^27^^–^37^,^^39^

*‘I mean, she’s just someone who can make you do things … Not as in a bad way, she sort of, like, empowers you, for want of a better word, to do it, you know what I mean?* [She motivates you] *“… give it a go,” and she’ll explain … and if you don’t go she doesn’t get disappointed or anything like that, she just says, “Oh, right, well, we’ll sort something else out for you.”’*^32^

Patients’ trust in social prescribing enhanced their willingness to seek help. The supportive nature of staff at social prescribing destinations (for example, organisations/groups in which the patients were connected to) further reinforced this trust;^22^^,^^23^^,^^27^^–^30^,^^32^^–^36^,^^39^^,^^40^ if individuals there were approachable, enquired about pertinent issues, and provided useful assistance:

*‘I know now that if there’s a problem I can text* [social café coordinator]*, I can go into the office and say look I’ve got this problem I don’t know how to handle it …’*^35^

It should be noted that some patients developed positive feelings for their link worker, calling them *‘a friend’*^26^ or *‘a pal’*.^39^ This could lead to a sense of grief when the social prescribing pathway ended and patients missed their link worker*.*^24^

### Misalignment producing no response

At times, non-clinical interventions recommended in social prescribing did not align with the patient’s needs or expectations, resulting in an absence of positive engagement or outcomes. This theme stressed the necessity of aligning recommended activities with patient preferences to ensure engagement and positive implications.

Severe mental health conditions negatively affected engagement;^23^^–^26^,^^29^^,^^31^^–^35^,^^38^^–^40 such conditions often overshadowed social prescribing’s potential benefit, underscoring a misalignment of approaches to address such complexities. Other challenges, such as poor living conditions and lack of a support system affected patients’ abilities to adopt and maintain positive practices from social prescribing:

*‘However, his poor living conditions and social isolation hindered positive practices and uptake of the “good advice” about diet he said he had received from the intervention.’*^33^

Certain patients encountered challenges because of significant familial and economic responsibilities; gender roles and socioeconomic status influenced how individuals prioritised their health and wellbeing. In deprived communities, male patients often prioritised work to meet economic needs.^25^ In contrast, female patients typically prioritised domestic responsibilities over personal health, particularly when lacking a support system, making social prescribing appointments feel like an additional burden rather than benefit:

*‘Sometimes I don’t take medication because I forgot because I’m so concentrated on the boys … the last thing I want is somebody to say* [to me]*, “You want to see me?” or appointment or I need to come to school, you know* [laughs]*? Even to cook.’*^28^

Furthermore, misalignment factors in social prescribing included a disconnect between the patient’s perceived needs and what link workers believed to be beneficial, logistical issues like timing or location of the prescribed activities, or cultural or personal preferences that were not adequately considered. As a result, patients were less likely to engage or benefit from the prescribed activities. Unmet expectations frequently led to distress. The role of hope in patients’ experiences with social prescribing was crucial; those who saw no response felt hopeless despite seeking help:^23^^,^^25^^,^^28^^,^^30^^,^^33^^,^^39^

*‘In this case, the participants highlight their disappointment in not feeling well treated or having their needs understood, especially after a lot of effort was required to make the first step (“leave the house”).’*^30^

## Discussion

### Summary

This thematic synthesis of patients’ experiences of social prescribing in the UK resulted in five analytical themes:

- searching for hope in times of adversity;
- variability in temporal responsiveness;
- sustained change from a positive response;
- feeling supported–encouraged; and
- misalignment producing no response.

This research supported the typology of a patient’s journey along the social prescribing pathway and highlighted that individual positive outcomes could extend beyond the person, fostering community resilience.

### Strengths and limitations

To the authors’ knowledge, this is the first thematic synthesis of patients’ experiences of social prescribing in the UK. This review involved four reviewers, which allowed for cross-checking and validation of data. Including only peer-reviewed journal articles ensured a high standard of data, but it is possible that some important information might have been omitted because grey literature can offer additional insights.^41^

Focusing on the UK provided an opportunity to examine social prescribing in context, considering the unique regulatory, cultural, and systemic factors shaping primary care in this country. This might limit how findings can be applied in other countries, although the author’s believe that themes will have wider resonance than just the UK.

### Comparison with existing literature

Findings from this review can be understood through the lens of two related theories: the transtheoretical model (TTM)^42^ and person-centred care (PCC).^43^ The theme ‘searching for hope in times of adversity’ highlighted a need for patients to find positivity and support, especially during challenging periods, leading them to seek help. PCC proposes that health care should focus on the whole person, not just their illness.^43^ When patients face difficult times, they often feel isolated and overwhelmed, seeking not only medical solutions but also a desire to be heard and understood.^22^^,^^24^^,^^25^^,^^27^^,^^33^^,^^34^ By listening to patients and involving them in their care decisions, we can give them a sense of control and value.^43^ When patients feel understood and involved, it can give them hope.^22^^–^24^,^^26^^–^39 This can start in primary care, which is the entry point to most social prescribing in the UK.^3^

The review also revealed various patient responses to social prescribing. TTM proposes that when individuals attempt to modify their health behaviours, they transition through distinct phases: precontemplation, contemplation, preparation, action, maintenance, and termination.^42^ This model incorporates processes and strategies to support transitions between stages, highlighting the importance of recognising an individual’s position in their change journey to deliver suitable interventions.^44^ For patients experiencing an immediate impact from social prescribing, they might already be in the action or preparation stages of the TTM. They are eager to make changes to their life and quickly benefit from the social prescribing pathway,^22^^–^27^,^^29^^–^36^,^^38^^,^^39^ highlighting their readiness and active engagement with new resources. In contrast, those experiencing a protracted impact could be in the earlier stages of the TTM, such as precontemplation or contemplation. Although they might recognise potential benefits, their inherent hesitation or ambivalence, combined with their unique life circumstances,^22^^,^^23^^,^^25^^,^^27^^,^^29^^,^^31^^–^40 could mean they need more time to fully embrace the changes. The longer timeframe for benefits highlights the personalised nature of health and wellbeing, emphasising the need for PCC in social prescribing. However, this may be challenging if link workers face time constraints in terms of how long they can spend supporting each patient.

Feeling supported and encouraged is crucial to patients’ experiences of social prescribing and aligns with the TTM’s focus on self-efficacy and supportive environments for facilitating change.^45^ Link workers in social prescribing provide a supportive environment similar to the ‘therapeutic alliance’ in counselling, fostering a collaborative and trustful bond that is crucial for positive outcomes.^46^^,^^47^ The initiation of a robust connection marked by active listening, empathy, and validation is integral.^22^^–^24^,^^26^^–^39 However, the evolving nature of the link worker–patient relationship, where some patients referred to link workers as *‘a friend’*^26^ or *‘a pal’*,^39^ raises essential questions regarding professional boundaries. Some patients reported feelings of loss when the social prescribing pathway concluded.^26^^,^^39^ Counselling has clear ethical boundaries for client–counsellor relationships whereas link worker guidelines for the therapeutic alliance are still developing because of the profession’s relative infancy.

### Implications for research and practice

This review suggested a potential need for an assessment to ascertain a patient’s readiness for social prescribing at the point of referral to a link worker. It also highlighted the need for clear ethical guidelines to define professional boundaries in the link worker–patient relationship, ensuring supportive yet professional interactions. A structured protocol for the termination phase is essential to prevent patients from feeling lost and confused at this point, providing them with closure and guidance on next steps. Awareness campaigns for patients and health providers are important to increase both enrolment to and engagement with social prescribing.^48^ Social prescribing aims to provide equitable, universal access, but preliminary findings suggest variations in referral and decline rates in primary care, particularly among patients with long-term conditions, ethnic minorities, and younger people.^49^ Attention is therefore required to provide opportunities that help individuals gain resources to improve their health, rather than assuming that they are equally inclined or have capacity to invest in this.^25^

Further research to help with developing guidelines around the link worker–patient relationship is warranted. Social prescribing’s termination process needs to be better understood, to help design a seamless transition, emphasising the progress made by a patient and suggesting future resources or interventions if needed. In addition, the potential positive ripple effect of the social prescribing pathway within the community needs to be investigated.

In conclusion, patients could benefit long term from social prescribing when it meets their needs and expectations. The findings emphasise the importance of aligning social prescribing referrals with patients’ willingness and capacity to participate. Thus, it is advised that healthcare providers assess whether patients are a good fit for social prescribing before making a referral.
